# New prospectives in spinal anaesthesia for urogenital tract surgery in geriatric patients

**DOI:** 10.1186/1471-2482-13-S1-A9

**Published:** 2013-09-16

**Authors:** Anna d’Elia, Francesca Grassia, Claudia Orabona, Maria Luisa Mingione, Lucia Marullo, Giuseppe Dimarzio, Biagio Lettieri

**Affiliations:** 1 Dept. of Anesth. Surgical and Emergency Sciences-Intensive Care Unit - II University of Naples, Italy

## Introduction

Spinal anaesthesia (SA) is the most commonly used anesthetic technique for transurethral resection of prostate (TURP) surgery in geriatric patient population[1]. Many geriatric patients have coexisting cardiac or pulmonary diseases, so it's very important to limit the distribution of the block to prevent the possible hemodynamic and pulmonary adverse effects. The purpose of this study is to compare the effectiveness of using intrathecal low dose bupivacaine-fentanyl combination with conventional dose prilocaine-fentanyl combination for day case TURP surgery in geriatric patient population[1]. We hypothesized that, using low dose bupivacaine-fentanyl combination provides shorter duration of block duration and postanesthesia care unit (PACU) stay with better hemodynamic stability than using conventional dose prilocaine-fentanyl combination.

## Methods

Tacking as subjects 50 patients ASA II-III evaluation, 70 years of age or older with homogeneous co-morbidity, scheduled for elective TURP surgery without controindications for spinal anaesthesia we conducted the study. Patients' demographic data and surgery durations were comparable in groups. Patients were divided into 2 groups. Group A(n=25) received 4 mg bupivacine 0.5% + 25 µg fentanyl and Group B(n=25) received 50 mg prilocaine 2% + 25 µg fentanyl intrathecal. This study has compared block quality and duration, postanesthesia care unit stay and adverse effects in two groups. Intraoperative monitoring consisted of heart rate, noninvasive blood pressure and oxygen saturation, which were recorded every 5 minutes. The highest dermatomal level of sensory block, the time to reach this level and the motor blockade at the time of reaching highest dermatomal level of sensory block were recorded. Postoperative follow-up was continued in PACU every 10 minutes until the patient was discharged. Criteria for discharge from PACU were: stable vital signs for > 30 minutes, orientation of patient to person, time and place, hemostasis of surgical area, absence of adverse effects, absence of pain, absence of nausea and vomiting, resolution of motor and sensory blocks. The primary endpoints of the study were comparing the duration of spinal block and duration of PACU stay. Secondary endpoint of the study was comparing the adverse effects like hypotension, bradycardia, PONV, block failure and pain during the operation. Failure to achieve a block level of T10 or additional analgesia request was considered as block failure. Hypotension was defined as a systolic blood pressure < 30% of preoperative value and bradycardia was defined as heart rate < 50.min^-1^. These adverse effects were treated by either bolus atropine and/or ephedrine.

## Results

Mean dermatomal level of highest sensorial block was higher in Group B(T8) than in Group A(T10). But the time to reach this highest sensorial block level were comparable in groups. Motor block at the time of reaching highest sensorial block in Group A was less than in Group B. Duration of block and PACU stay were significantly shorter in Group A (100.2 than in Group B(145,3. ± 10.8). Adverse effects during the procedure were shown (Table 1). In Group B, hypotension was seen in 4 patients and bradycardia was seen in 5 patients. Only one patient in Group A had hypotension and bradycardia. PONV(postoperative nausea and vomiting) was detected in one patient in Group A, and in six patients of Group B. These differences were significant between groups. None of the patients in either groups manifested block failure or pain during the procedure.

**Table 1:** Adverse effects in two groups

Adverse effects	Group A(n=25)	Group B (n=25)
Bradycardia	1	5

Hypotension	1	4

PONV	1	6

Block failure	0	0

Pain during procedure	0	0

## Conclusions

As known, systemic hypotension and bradycardia are the most common side effects during central neural blocks. Marked hypotension can be deleterious especially in geriatric patients with limited cardiac reserve[2,3]. High incidence of coronary disease in geriatric patients increases the risk of myocardial ischemia due to hypotension[2]. A high level of block is another important factor in the development of hypotension during SA[4]. Prilocaine was reported as such in day case surgeries with low incidence of transient neurological symptoms TNS. However, marked hypotension and bradycardia were reported in intrathecal prilocaine use. Bupivacaine has a low risk of TNS as well. Nonetheless, if used in conventional doses in day case surgeries, its main disadvantages are long duration of action and recovery and hemodynamic adverse effects like hypotension. Many different attempts have been attempted to decrease the block duration of bupivacaine, like lowering the dose and adding adjuvant drugs. Intrathecal opioids are known to enhance analgesia of subtherapeutic doses of local anesthetics. Thus, successful SA can be achieved by combining intrathecal opioids with low doses of local anesthetics that would be inadequate when used independently[2]. Using low doses of local anesthetics could shorten the block duration and its recovery and could also prevent the undesired hemodynamic adverse effects.

In conclusion, adequate SA can be provided by using 4 mg bupivacaine and 25 µg fentanyl combination with shorter block duration and PACU stay when compared with 50 mg prilocaine and 25 µg fentanyl combination for day case TURP surgeries. We also observed that a stable hemodynamic profile with low-dose bupivacaine becomes advantageous which is especially very important in geriatric patient population.

## References

[B1] Kararmaz A Kaya S Turhanoglu S Ozyılmaz MA Low dose bupivacaine-fentanyl spinal anesthesia for transurethral prostatectomy Anaesthesia 2003 13 526 530 10.1046/j.1365-2044.2003.03153.x 12846615

[B2] Kuusniemi KS Pihlajamaki KK Pitkanen MT Helenius HY Kirvela OA The use of bupivacaine and fentanyl for spinal anesthesia for urologic surgery Anesth Analg 2000 13 1452 1456 10.1097/00000539-200012000-00029 11093999

[B3] Rooke GA Freund PR Jacobson AF Hemodynamic response and change in organ blood volume during spinal anesthesia in elderly men with cardiac disease Anesth Analg 1997 13 99 105 9212130 10.1097/00000539-199707000-00018

[B4] Erdil F Bulut S Demirbilek S Gedik E Gulhas N Ersoy MO The effects of intrathecal levobupivacaine and bupivacaine in the elderly Anaesthesia 2009 13 942 946 10.1111/j.1365-2044.2009.05995.x 19686477

